# Supplementary material for: Chemical Profile of the Volatile Constituents and Antimicrobial Activity of the Essential Oils from Croton adipatus, Croton thurifer, and Croton collinus
Source: Antibiotics (Basel). 2021 Nov 12;10(11):1387. doi: 10.3390/antibiotics10111387 (PMC8614731; doi:10.3390/antibiotics10111387)
Supplement: Supplementary file 1 [file antibiotics-10-01387-s001.zip › antibiotics-1447802-supplementary.pdf]

Supplementary material

Chemical Profile of the Volatile Constituents and Antimicrobial Activity of the Essential Oils from *Croton adipatus*, *Croton thurifer*, and *Croton collinus*

Juana Liz Cucho-Medrano [1], Sammy Wesley Mendoza-Beingolea [1], César Máximo Fuertes-Ruitón [2], María Elena Salazar-Salvatierra [3], and Oscar Herrera-Calderón [4,*]

1 Faculty of Pharmacy and Biochemistry, Universidad Nacional Mayor de San Marcos, Jr. Puno 1002, Lima 15001, Peru; liz.cumed@gmail.com (J.L.C.-M.), sammy_w20@outlook.com (S.W.M-B.)
2 Institute for Research in Pharmaceutical Sciences and Natural Resources, Faculty of Pharmacy and Biochemistry, Universidad Nacional Mayor de San Marcos, Jr. Puno 1002, Lima 15001, Peru; cfuertesr@unmsm.edu.pe
3 Research Institute in Biological Chemistry, Microbiology and Biotechnology, Faculty of Pharmacy and Biochemistry, Universidad Nacional Mayor de San Marcos, Jr. Puno 1002, Lima 15001, Peru; msalazars@unmsm.edu.pe;
4 Department of Pharmacology, Bromatology and Toxicology, Faculty of Pharmacy and Biochemistry, Universidad Nacional Mayor de San Marcos, Jr. Puno 1002, Lima 15001, Peru. oherreraca@unmsm.edu.pe

* Correspondence: oherreraca@unmsm.edu.pe; Tel.: (+51-956-550-510)

Supplementary material	Pag.
**Figure S1. GC-MS chromatogram of the essential oil of *Croton adipatus* Kunth.**	3
**Figure S2. Spectrum of the main constituents of the essential oil of *Croton adipat*us Kunth. A: β-myrcene; B: α-thujene; C: D-Limonene.**	3
**Figure S3. GC-MS chromatogram of the essential oil of *Croton thurifer* Kunth.**	3
**Figure S4. Spectrum of the main constituents of the essential oil of *Croton thurifer* Kunth. A: Unknown ($C_{10}H_{16}$); B: Unknown ($C_{15}H_{26}O$); C: β-elemene.**	4
**Figure S5. GC-MS chromatogram of the essential oil of *Croton collinus* Kunth.**	4
**Figure S6. Spectrum of the main constituents of the essential oil of *Croton collinus* Kunth. A: β-caryophyllene B: D-limonene; C: β-thujene.**	4
**Table S1. Physicochemical properties of the essential oils of Croton species.**	5
**Table S2. Retention times of the chemical compounds of *Croton adipatus* Kunth essential oil.**	6

**Table S3. Retention times of the chemical compounds of *Croton thurifer* Kunth essential oil.**	8
**Table S4 Retention times of the chemical compounds of *Croton collinus* Kunth essential oil.**	9
**Figure S7. Determination of the Minimum Inhibitory Concentration of the standard drugs. SC (Sterility Control), GC (Growth Control), Sa (S. aureus), Bs (B. subtilis), Ec (E. coli), Pa (P. aeruginosa), Ca (C. albicans).**	10

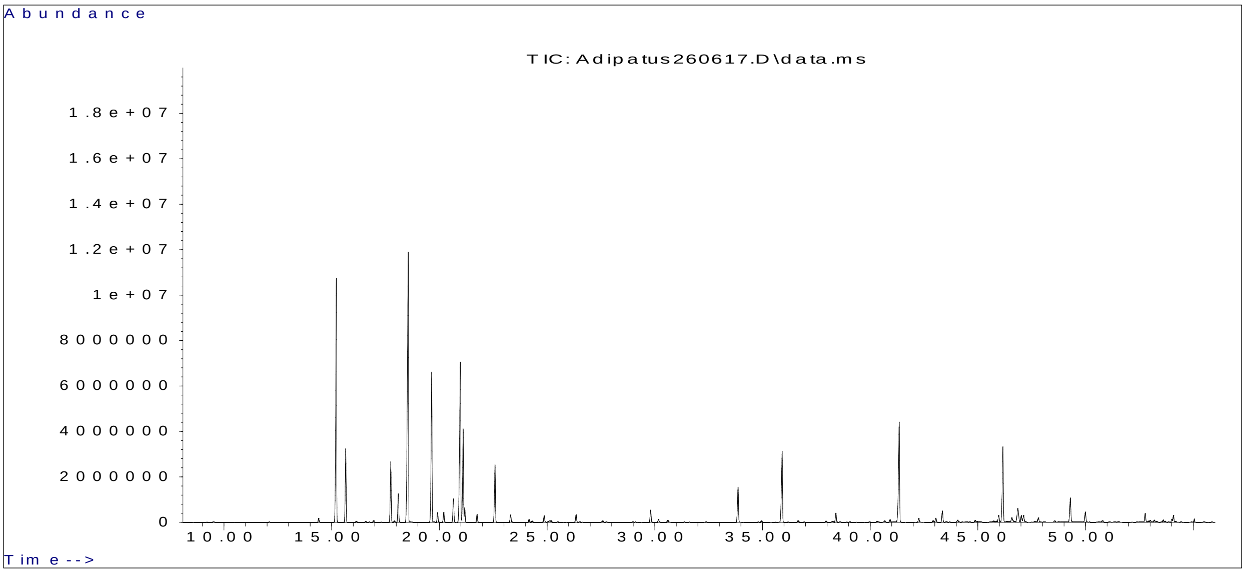

Figure S1. GC-MS chromatogram of the essential oil of *Croton adipatus* Kunth.

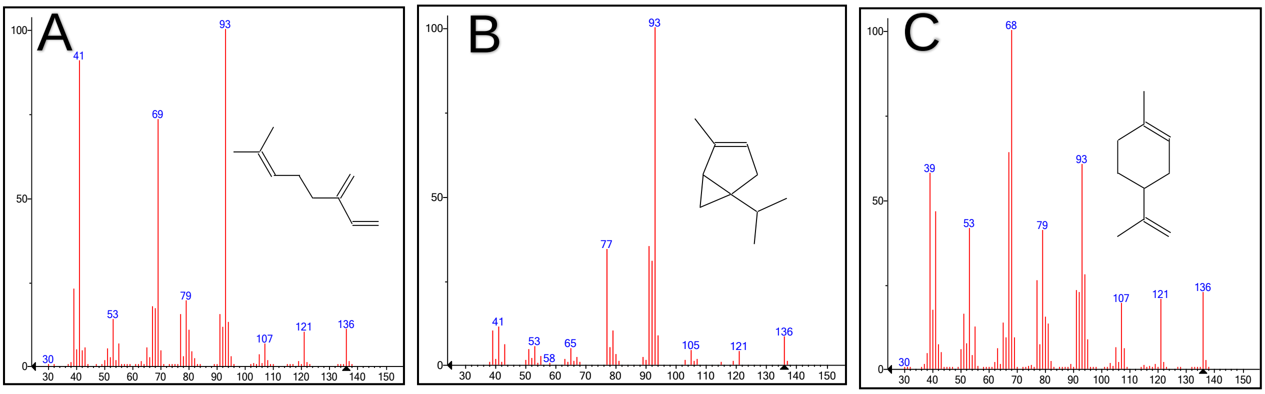

Figure S2. Spectrum of the main constituents of the essential oil of *Croton adipatus* Kunth. A: β-myrcene; B: α-thujene; C: D-Limonene.

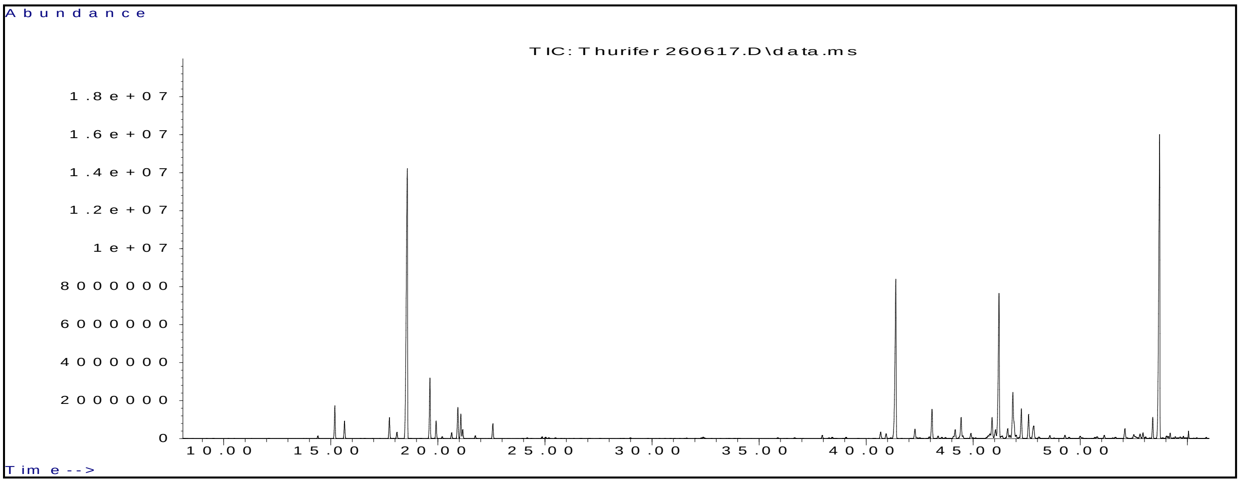

Figure S3. GC-MS chromatogram of the essential oil of *Croton thurifer* Kunth.

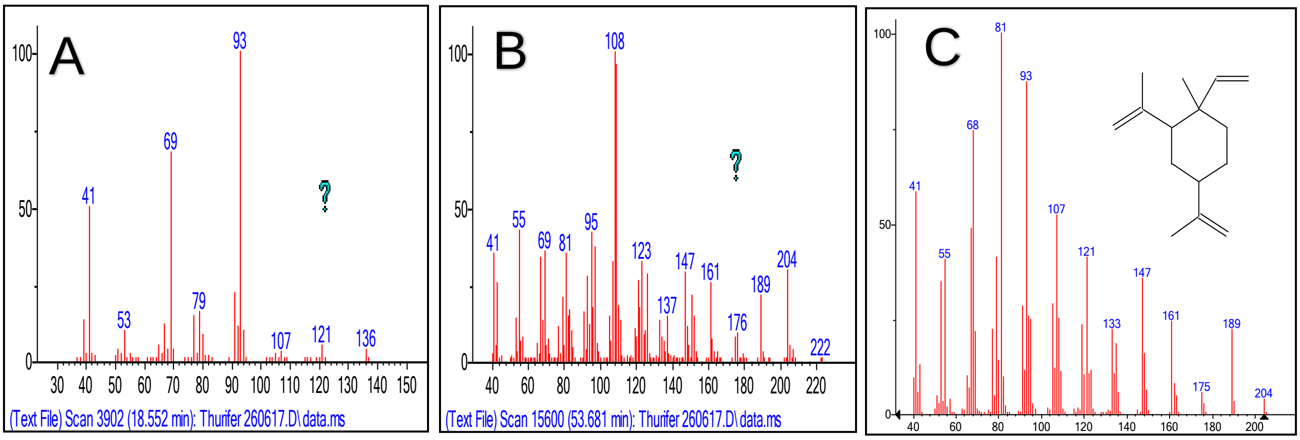

Figure S4. **Spectrum of the main constituents of the essential oil of *Croton thurifer* Kunth. A: Unknown ($C_{10}H_{16}$); B: Unknown ($C_{15}H_{26}O$); C: β-elemene.**

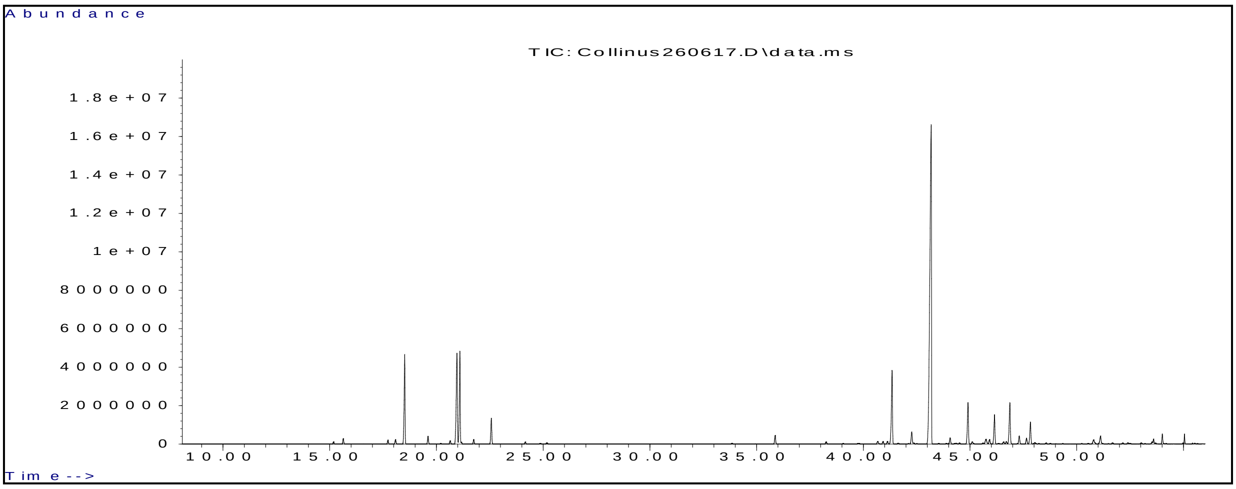

Figure S5. GC-MS chromatogram of the essential oil of *Croton collinus* Kunth.

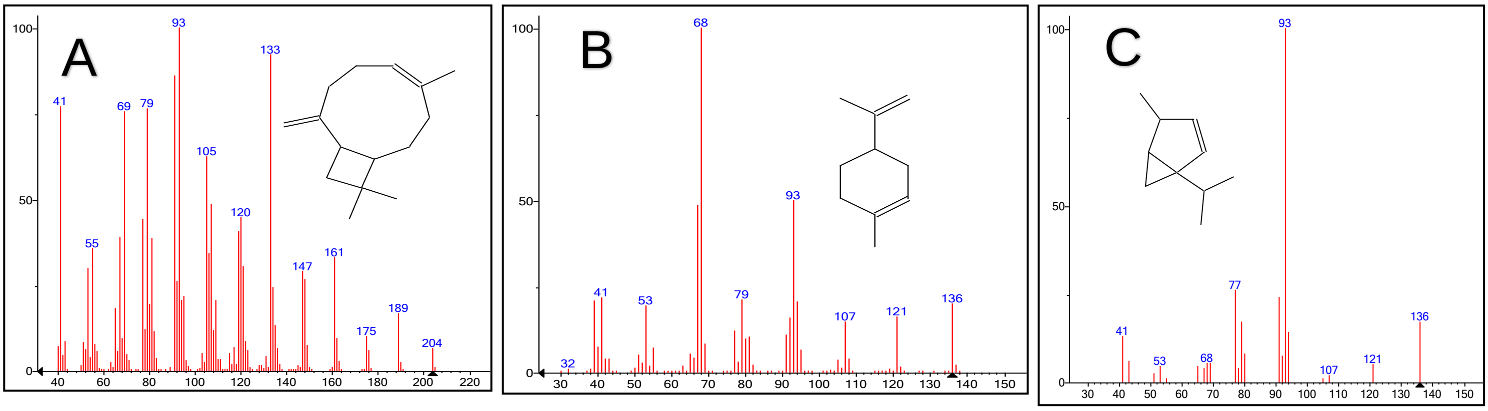

Figure S6. Spectrum of the main constituents of the essential oil of *Croton collinus* Kunth. A: β-caryophyllene B: D-limonene; C: β-thujene.

Table S1. Physicochemical properties of the essential oils of Croton species.

	***Croton adipatus* Kunth**	***Croton thurifer* Kunth**	***Croton collinus* Kunth**
**Solubility**	Insoluble in water and ethanol 50%, soluble in methanol, DMSO and absolute ethanol.	Insoluble in water and ethanol 50 %, soluble in methanol, DMSO and absolute ethanol.	Insoluble in water and ethanol 50 %, soluble in methanol, DMSO and absolute ethanol.
**Density (25°C)**	0.9064 g/mL	0.9371 g/mL	0.9117 g/mL
**Refractive index (20°C)**	1.4715	1.4800	1.4845
**Yield (%)**	0.47± 0.01	0.07± 0.005	0.06 ± 0.001

Table S2. Chemical composition of *Croton adipatus* Kunth essential oil.

N°	Chemical constituents	Rt (min)
1	Isobutyl isobutyrate	14.39
2	α-thujene	15.22
3	α-Pinene	15.65
4	Sabinene	17.74
5	β-Pinene	18.09
6	β-Myrcene	18.55
7	α-Phellandrene	19.65
8	Isoamyl isobutyrate	19.92
9	α-Terpinene	20.20
10	o-Cymene	20.65
11	D-Limonene	20.97
12	β-Phellandrene	21.11
13	Eucalyptol	21.18
14	cis-Ocimene	21.75
15	τ-Terpinene	22.58
16	β-Terpineol	23.31
17	Terpinolene	24.16
18	β-Linalool	24.87
19	1,6-Dimethylhepta-1,3,5-triene	26.35
20	4-Terpineol	29.81
21	2-Decanone	30.17
22	α-Terpineol	30.60
23	2-Undecanone	33.87
24	2-Dodecanone	35.91
25	2-Carene	38.41
26	Unknown ($C_{15}H_{24}$)	40.92
27	β-Elemene	41.35
28	2-Ethyl-1,3-dimethyl-Benzene	42.26
29	β-Caryophyllene	43.05
30	δ-Elemene	43.35
31	Nerylacetone	44.07
32	α-Muurolene	45.97
33	Germacrene D	46.17
34	α-Selinene	46.58
35	Bicyclogermacrene	46.86
36	Geranil isobutyrate	47.02
37	δ-Cadinene	47.12
38	Unknown ($C_{15}H_{24}$)	47.81
39	Elemol	49.29
40	Unknown ($C_{15}H_{24}$)	49.99
41	Unknown ($C_{15}H_{26}O$)	52.77
42	β-Maaliene	53.19

43	Unknown (C_{15}H26O)	53.60
44	τ-Muurolol	54.01
45	Unknown ($C_{15}H_{26}O$)	54.08
46	Heptadecane	55.05

Table S3. Chemical composition of *Croton thurifer* Kunth essential oil.

N°	Chemical constituents	Rt (min)
1	α-Thujene	15.19
2	α-Pinene	15.64
3	Sabinene	17.74
4	β-Pinene	18.09
5	Unknown ($C_{10}H_{16}$)	18.57
6	α-Phellandrene	19.63
7	Isopentyl isobutyrate	19.92
8	p-Cymene	20.64
9	D-Limonene	20.93
10	β-Phellandrene	21.07
11	Eucalyptol	21.16
12	1-methyl-4-(1-methylethyl)-1,4-cyclohexadiene	22.57
13	Copaene	40.68
14	1-ethenyl-1-methyl-2,4-bis (1-methylethenyl)-cyclohexane	40.93
15	β-Elemene	41.39
16	Cyperene	42.28
17	β-Caryophyllene	43.08
18	Unknown ($C_{15}H_{24}$)	44.16
19	L-Alloaromadendrene	44.44
20	α-Caryophyllene	44.89
21	α-Elemene	45.77
22	Unknown ($C_{15}H_{24}$)	45.88
23	Unknown ($C_{15}H_{24}$)	46.04
24	Germacrene D	46.21
25	Eremophilene	46.61
26	Unknown ($C_{15}H_{26}O$)	46.85
27	Unknown ($C_{15}H_{26}O$)	47.59
28	δ-Cadinene	47.83
29	Unknown ($C_{15}H_{26}O$)	52.08
30	Unknown ($C_{15}H_{22}O$)	52.93
31	Unknown ($C_{9}H_{14}O$)	53.39
32	Unknown ($C_{15}H_{26}O$)	53.70
33	Unknown ($C_{15}H_{26}O$)	54.20
34	Heptadecane	55.06

Table S4. Chemical composition of *Croton collinus* Kunth essential oil.

N°	Chemical constituents	Rt (min)
1	α-Thujene	15.18
2	α-Pinene	15.64
3	Sabinene	17.73
4	β-Pinene	18.08
5	β-Myrcene	18.51
6	α-Phellandrene	19.61
7	p-Cymene	20.64
8	D-Limonene	20.96
9	β- Thujene	21.10
10	cis-Ocimene	21.75
11	1-methyl-4- (1-methylethyl) -1,4-cyclohexadiene	22.57
12	Terpinolene	24.16
13	2-Undecanone	35.87
14	1,5,5-Trimethyl-6-methylene-cyclohexene	38.25
15	Copaene	40.68
16	Unknown ($C_{15}H_{24}$)	40.93
17	Unknown ($C_{15}H_{24}$)	41.13
18	β-Elemene	41.35
19	Cyperene	42.27
20	β-Caryophyllene	43.18
21	Geranil acetone	44.07
22	α-Caryophyllene	44.91
23	Aromadendrene	45.10
24	Valencene	45.74
25	α-Curcumene	45.91
26	Germacrene D	46.15
27	α-Selinene	46.57
28	β-Cubebene	46.70
29	Bicyclogermacrene	46.87
30	Unknown ($C_{15}H_{24}$)	47.31
31	α-Muurolene	47.65
32	δ-Cadinene	47.83
33	(-)-Spatulenol	50.79
34	Unknown ($C_{15}H_{24}O$)	51.12
35	τ-Muurolene	53.53
36	τ-Muurolol	53.60
37	α-Cadinol	54.01
38	Heptadecane	55.05

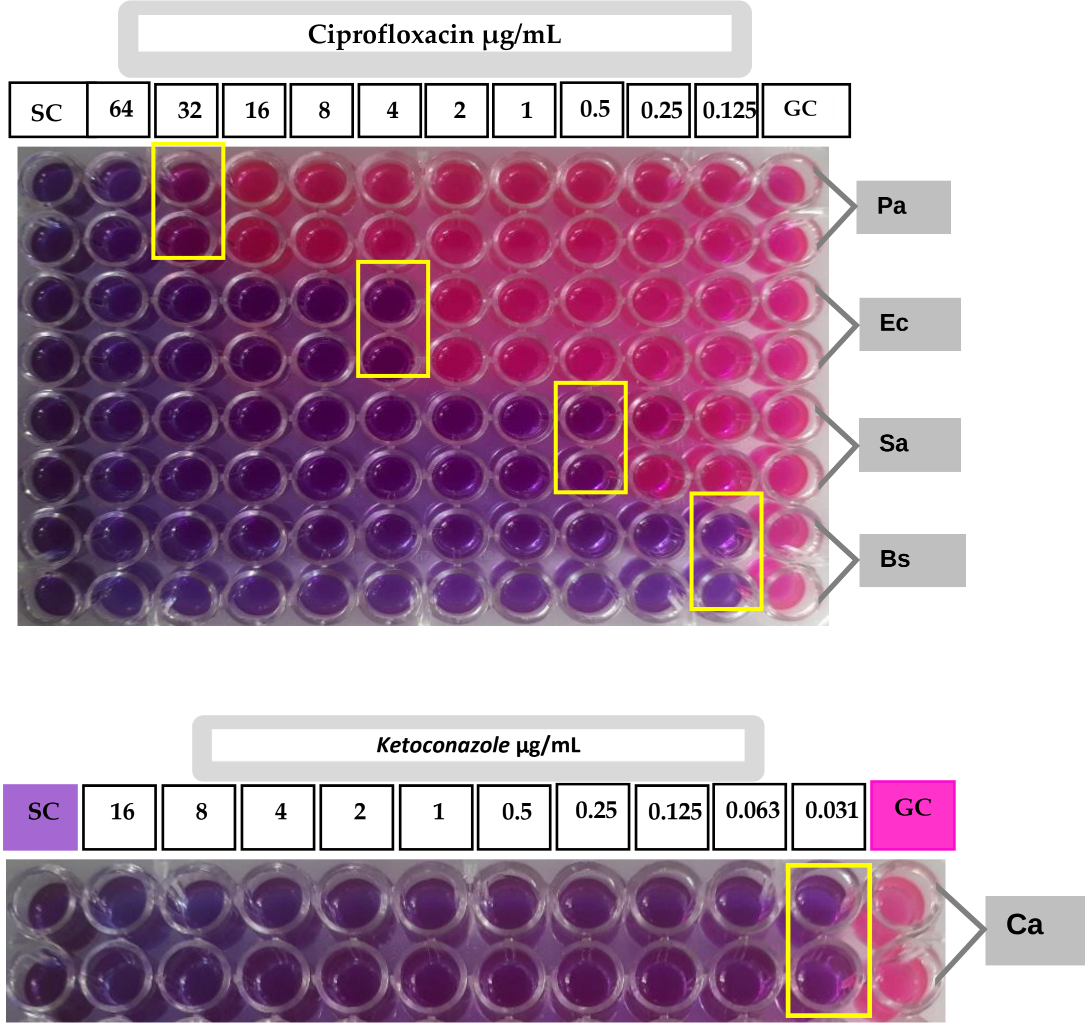

Figure S7. Determination of the Minimum Inhibitory Concentration of the standard drugs. SC (Sterility Control), GC (Growth Control), Sa (*S. aureus*), Bs (*B. subtilis*), Ec (*E. coli*), Pa (*P. aeruginosa)*, Ca (*C. albicans*).
